# The Fungal Metabolite (+)-Terrein Abrogates Ovariectomy-Induced Bone Loss and Receptor Activator of Nuclear Factor-κB Ligand–Induced Osteoclastogenesis by Suppressing Protein Kinase-C α/βII Phosphorylation

**DOI:** 10.3389/fphar.2021.674366

**Published:** 2021-06-08

**Authors:** Kyosuke Sakaida, Kazuhiro Omori, Masaaki Nakayama, Hiroki Mandai, Saki Nakagawa, Hidefumi Sako, Chiaki Kamei, Satoshi Yamamoto, Hiroya Kobayashi, Satoki Ishii, Mitsuaki Ono, Soichiro Ibaragi, Keisuke Yamashiro, Tadashi Yamamoto, Seiji Suga, Shogo Takashiba

**Affiliations:** ^1^ Department of Pathophysiology-Periodontal Science, Graduate School of Medicine, Dentistry and Pharmaceutical Sciences, Okayama University, Okayama, Japan; ^2^ Department of Periodontics and Endodontics, Okayama University Hospital, Okayama, Japan; ^3^ Department of Oral Microbiology, Graduate School of Medicine, Dentistry and Pharmaceutical Sciences, Okayama University, Okayama, Japan; ^4^ Department of Pharmacy, Faculty of Pharmacy, Gifu University of Medical Science, Gifu, Japan; ^5^ Division of Applied Chemistry, Graduate School of Natural Sciences and Technology, Okayama University, Okayama, Japan; ^6^ Department of Molecular Biology and Biochemistry, Graduate School of Medicine, Dentistry and Pharmaceutical Sciences, Okayama University, Okayama, Japan; ^7^ Department of Oral Maxillofacial Surgery, Graduate School of Medicine, Dentistry and Pharmaceutical Sciences, Okayama University, Okayama, Japan

**Keywords:** (+)-terrein, ovariectomy, osteoporosis, RANKL, PKC

## Abstract

Osteoporosis is a common disease characterized by a systemic impairment of bone mass and microarchitecture that results in fragility fractures. Severe bone loss due to osteoporosis triggers pathological fractures and consequently decreases the daily life activity and quality of life. Therefore, prevention of osteoporosis has become an important issue to be addressed. We have reported that the fungal secondary metabolite (+)-terrein (TER), a natural compound derived from *Aspergillus terreus*, has shown receptor activator of nuclear factor-κB ligand (RANKL)–induced osteoclast differentiation by suppressing nuclear factor of activated T-cell 1 (NFATc1) expression, a master regulator of osteoclastogenesis. TER has been shown to possess extensive biological and pharmacological benefits; however, its effects on bone metabolism remain unclear. In this study, we investigated the effects of TER on the femoral bone metabolism using a mouse-ovariectomized osteoporosis model (OVX mice) and then on RANKL signal transduction using mouse bone marrow macrophages (mBMMs). *In vivo* administration of TER significantly improved bone density, bone mass, and trabecular number in OVX mice (*p* < 0.01). In addition, TER suppressed TRAP and cathepsin-K expression in the tissue sections of OVX mice (*p* < 0.01). In an *in vitro* study, TER suppressed RANKL-induced phosphorylation of PKCα/βII, which is involved in the expression of NFATc1 (*p* < 0.05). The PKC inhibitor, GF109203X, also inhibited RANKL-induced osteoclastogenesis in mBMMs as well as TER. In addition, TER suppressed the expression of osteoclastogenesis-related genes, such as *Ocstamp*, *Dcstamp*, *Calcr*, *Atp6v0d2*, *Oscar*, and *Itgb3* (*p* < 0.01). These results provide promising evidence for the potential therapeutic application of TER as a novel treatment compound against osteoporosis.

## Introduction

At present, more than 15 million people have osteoporosis, which is a serious problem in Japan, where the population is extremely aged (Hagino et al., 2021). In the United States, there were more than 2 million osteoporosis-related fractures in 2005, with associated costs of approximately $ 17 billion. By 2023, the annual number of fractures will exceed 3 million, and the U.S. healthcare system is projected to cost $ 25 billion (Burge et al., 2007). Osteoporosis is caused by a deficiency of female hormone estrogen, which breaks bone homeostasis between osteoclasts and osteoblasts. Severe bone loss due to osteoporosis triggers pathological fractures and, consequently, decreases daily life activity and the quality of life. Therefore, the prevention of osteoporosis is one of the most urgent issues in Japan (Tilman et al., 2011; Katrin, 2019).

Currently, bisphosphonates (BPs) are mainly used in the treatment of osteoporosis (Shibahara et al., 2018). However, medicine-induced osteonecrosis of the jaw (MRONJ), which occurs after invasive dental procedures, such as tooth extraction, scaling, and root planning, has become a major problem in the treatment of osteoporosis, including BP treatment (Yoneda et al., 2017). The incidence of MRONJ in Japan is 0.85 per 100,000 people per year (Yoneda et al., 2017). According to a nationwide survey by the Japanese Society of Oral and Maxillofacial Surgery, it is estimated to be approximately 0.01–0.02% (Yoneda et al., 2010). In Japan, the number of remaining teeth in the elderly continues to increase as a result of the 8,020 movement (Nasu and Nakamura, 2016). Therefore, since MRONJ significantly decreases the quality of life of patients, it is desirable to develop new osteoporosis drugs without serious side effects.

**FIGURE 1:**
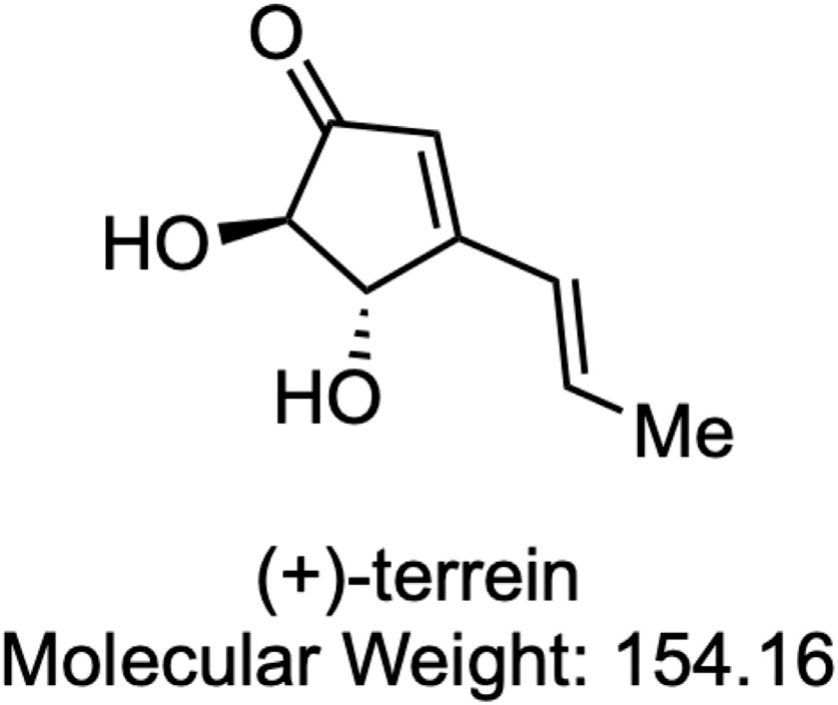
Chemical structure of (+)-terrein. Me: methyl.

Our research group has identified (+)-terrein (TER, Figure 1) as a naturally occurring compound with a different mechanism of action from that of molecularly targeted drugs, which is expected to have an anti-inflammatory effect by inhibiting interleukin (IL)-6 (Mandai et al., 2014). TER is a small molecule with a molecular weight of 154.16 kDa isolated from *Aspergillus terreus* as a secondary metabolite (Raistrick and Smith, 1935). TER has been reported to have various biological effects, such as inhibiting biofilm formation (Kim et al., 2018), angiogenin secretion in prostate cancer cells (Arakawa et al., 2008), and pulp inflammation (Lee et al., 2008). In addition, our group has established a route for the organic synthesis of TER (Mandai et al., 2014). Furthermore, this synthesized TER inhibits IL-6–induced vascular endothelial growth factor (VEGF) secretion in human gingival fibroblasts (Mandai et al., 2014). In addition, synthetic TER suppressed IL-6–induced macrophage colony-stimulating factor (M-CSF) secretion by inhibiting Janus-activated kinase (JAK)-1 phosphorylation (Yamamoto et al., 2018). We have also reported that TER has an inhibitory effect on RANKL or tumor necrosis factor (TNF)-α–induced osteoclastogenesis by suppressing the expression of nuclear factor of activated T-cell c1 (NFATc1), a master regulator of osteoclast differentiation in mouse bone marrow macrophages (mBMMs) (Nakagawa et al., 2020). In addition, we also showed that TER did not suppress RANKL-induced phosphorylation of NF-κB and MAPKs (ERK1/2 and p38), which are related to NFATc1 expression, meaning which it is still unknown about the effects of TER on RANKL signaling (Nakagawa et al., 2020). Based on these *in vitro* findings, we hypothesized that TER could suppress bone resorption in the femur if its anti-inflammatory and anti-osteoclastogenic effects were effectively exerted in the bones. However, the *in vivo* effect of TER on bone destruction remains unknown. In addition, to the best of our knowledge, there have been no studies on whether TER also acts on osteoblasts and whether it has an effect on bone homeostasis.

In this study, we evaluated the effect of TER on bone homeostasis *in vivo* using a mouse ovariectomy model of osteoporosis (OVX mice). We also assessed the effects of TER on the signaling pathways of RANKL-induced osteoclast differentiation using mBMMs *in vitro*, especially focused on protein kinase-C cascade which is also related to NFATc1 expression (Shin et al., 2014). PKC family members phosphorylate a wide variety of protein targets and are known to be involved in diverse cellular signaling pathways. In RANKL-induced osteoclast formation, PKC β functions, but not PKCδ, occur through glycogen synthase kinase-3β (GSK-3β)–NFATc1 axis (Shin et al., 2014). We also assessed the effects of TER on osteoblast differentiation using MC3T3-E1 cells *in vitro*. Our results suggest that host modulation using TER could be a novel therapeutic modality for the prevention of bone resorption, including osteoporosis.

## Materials and Methods

### Reagents

TER was synthesized from dimethyl l-tartrate. All spectra, including ^1^H and ^13^C nuclear magnetic resonance (NMR) spectra, infrared (IR) spectra, and specific rotation of the synthetic TER, were similar to those of natural TER and those that were previously described (Mandai et al., 2014). The following were used: human soluble RANKL (Wako, Hiroshima, Japan), human M-CSF (Leucoprol, Kyowa Hakko Kogyo, Japan), zoledronic acid (ZOL: Tokyo Chemical Industry, Japan), rabbit anti-total PKCα, rabbit anti–phospho-PKCα/βII and PKCδ monoclonal antibodies (Cell Signaling Technology Danvers, MA, United States), mouse anti–β-actin monoclonal antibody (Sigma-Aldrich, St. Louis, MO, United States), and the PKC inhibitor GF109203X (Bisindolylmaleimide I, Selleckchem, Japan).

### The Ovariectomized Mice Model

**FIGURE 2:**
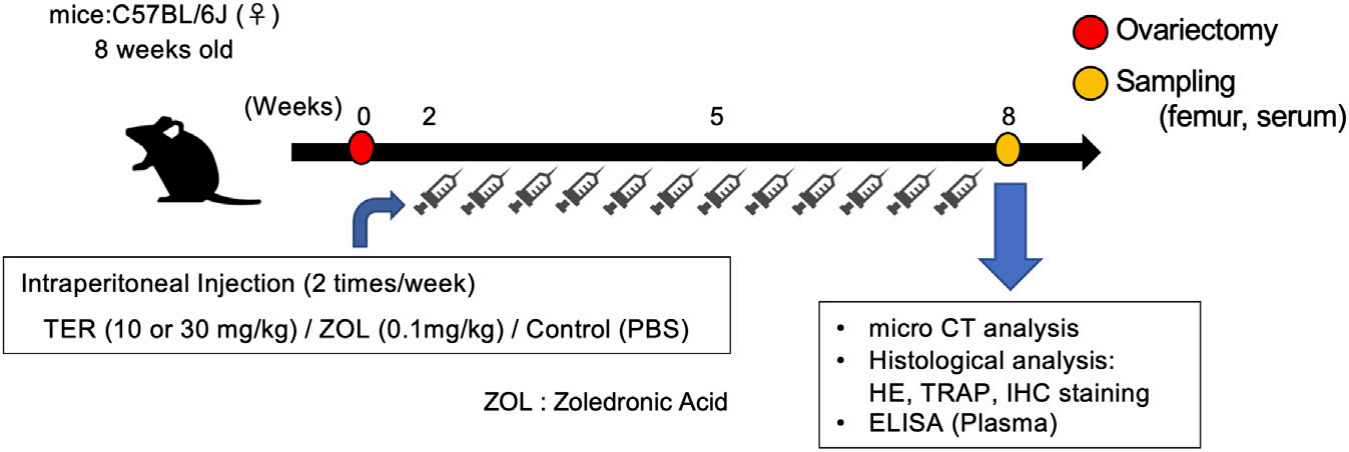
Schedule of the animal experiments. Eight-week-old C57BL/6 mice underwent ovariectomy and were randomly divided into four groups. Two weeks after ovariectomy, the mice were treated with (1) TER (10 mg/kg), (2) TER (30 mg/kg), and (3) PBS (200 μL) as a negative control, and (4) zoledronic acid (0.1 mg/kg) as a positive control; human dose was converted to mouse weight. Intraperitoneal injections were administered twice a week. The Sham group was sham-operated and administered PBS. Eight weeks after ovariectomy, the mice were sacrificed, and the femur bones and serum were collected.

Female C57BL/6J mice were obtained from Japan CLEA (Japan) and allowed to acclimatize for 1 week. At 8 weeks of age, mice were randomly divided into four groups (*n* = 6–7 per group): 1) Sham, 2) OVX, 3) OVX + TER (10–30 mg/kg, two times a week), and 4) OVX + ZOL (0.1 mg/kg, two times a week). A fake operation was performed in the Sham group as a control, and the other three groups underwent bilateral OVX under anesthesia with sodium pentobarbital (60 mg/kg) *via* intraperitoneal injection (Thompson et al., 1995; Xiao et al., 2018; Xu et al., 2018). The Sham and OVX groups received equal doses of phosphate-buffered saline (PBS) as controls, the OVX + TER group was given an intraperitoneal injection of TER at the indicated concentrations, and the OVX + ZOL group received an intraperitoneal injection of ZOL at the indicated concentrations after surgery. After 8 weeks, all mice were euthanized *via* cardiac stick exsanguination. Serum samples were prepared and stored at −80°C for the measurement of tumor necrosis factor (TNF)-α, IL-1β, IL-6, and M-CSF. The femurs of the mice were collected. This animal experiment was performed in accordance with the Guidelines for Proper Conduct of Animal Experiments of the Science Councils of Japan and approved by the Animal Research Control Committee of Okayama University (approval no: OKU-2018756), and the mice were kept under SPF conditions (Figure 2).

### Image Analysis Using Micro-Computed Tomography

To observe the femur bone resorption, 4-μm-thick slices from the mice from each group were photographed using an animal micro-CT imaging device (Skyscan: Bruker, Kontich, Belgium), and these images were reconstructed using the accompanying software (Reconstruction software, NRecon: Bruker) and analyzed using the image processing software (Analyze software, CT-Analyzer: Bruker). We measured the indicators of bone mineral density (BMD, mg/cm^3^), bone volume/trabecular volume (BV/TV, %), trabecular number (Tb.N, 1/μm), and trabecular separation (Tb.Sp, μm).

### Histological Analysis

The femur samples were fixed in a 4% paraformaldehyde solution (pH 7.4; Wako Pure Chemicals, Osaka, Japan) for 1 day. The blocks were immersed in disodium ethylenediaminetetraacetic acid (10% EDTA 2Na solution, pH 7.0, Muto Chemical Co., Ltd., Tokyo, Japan) for 10 days for demineralization, and were dehydrated using an ethanol series. Paraffin-embedded blocks were sliced into 4-μm-thick sections. Subsequently, histopathological analysis was performed using the following staining methods.

#### Hematoxylin-Eosin Staining

The prepared paraffin sections were subjected to HE staining, according to the normal method, and then encapsulated using a cover glass and Mount-Quick (Daido Sangyo Co., Ltd., Tokyo, Japan). Histological images were then observed under an optical microscope (BX-50; Olympus, Osaka, Japan).

#### Tartrate-Resistant Acid Phosphatase Staining

The prepared paraffin sections were stained with a TRAP staining solution (Cosmo Bio Co., Ltd., Tokyo, Japan) according to the conventional method, and then encapsulated with a cover glass and Mount-Quick Aqueous medium. Histological images were then observed under an optical microscope. The number of TRAP-positive cells that existed within 3 mm just below the growth plate and that had at least three nuclei were counted as osteoclasts. The number of osteoclasts was measured in nine sections from three mice in each group, and three sections from each mouse were selected at random.

#### Immunohistochemical Staining

The prepared paraffin sections were deparaffinized, rehydrated, and stained using the avidin–biotin peroxidase system and the Vectastain Elite ABC rat kit (Vector Laboratories, Burlingame, CA, United States). Tissue sections were immersed in a 0.3% hydrogen peroxide in methanol solution for 30 min at room temperature to remove the endogenous peroxidase activity and treated with trypsin (Thermo Fisher Scientific) for 15 min for antigen activation. After blocking with normal rabbit serum for 15 min, the primary antibodies were added and reacted overnight at 4°C. For the primary antibodies, anti–cathepsin-K and anti-alkaline phosphatase antibodies (Abcam, Cambridge, MA, United States), which are specific marker proteins of osteoclasts and osteoblasts, were diluted (100-fold) in PBS; they were washed in PBS, and rabbit anti-rat biotin-labeled secondary antibodies were added by diluting (200-fold) in PBS and were left to react for 30 min at 25°C. The avidin–biotin–labeled enzyme complex was added and reacted for 30 min, followed by the addition of 0.01% 3,3′-diaminobenzidine (DAB; Nacalai Tesque Co., Ltd., Kyoto, Japan) for coloration. Finally, cell nuclei were contrast-stained with Mayer's hematoxylin (Wako), observed after enclosure using a cover glass and Mount-Quick, and the positive cells were counted.

### Enzyme-Linked Immunosorbent Assay

Fresh blood was collected from the hearts of mice in each group. The serum was collected *via* centrifugation at 10,000 × *g* at 4°C for 15 min. The secretion of serum inflammatory cytokines (IL-1β, IL-6, TNF-α, and M-CSF) was measured using ELISA MAX Deluxe (BioLegend, San Diego, CA, United States) and Quantikine ELISA Kit (R&D systems, Minneapolis, MN, United States), following the manufacturer’s instructions. The absorbance at 450 nm was measured using a microplate reader (SH-1000 Lab; Corona Electric Co., Hitachinaka, Japan). All tests were performed in two wells per individual. The serum concentrations of IL-1β, IL-6, TNF-α, and M-CSF were quantified, and the concentrations were set to 0 if the results were below the detection limit.

### Cell Culture

#### Mouse Bone Marrow Macrophages

Five-week-old male C57BL6/J mice were obtained from Japan CLEA (Japan). Bone marrow cells were collected from the tibiae and femur, and were cultured in M-CSF (50 ng/ml) for 3 days in culture dishes containing DMEM (Wako) supplemented with 10% fetal bovine serum (FBS, Invitrogen, Carlsbad, CA, United States). After 3 days, floating cells were removed by rinsing with phosphate-buffered saline (PBS), and the attached cells were used as mBMMs (Horibe et al., 2013; Tevlin et al., 2014). Attached mBMMs were sprayed with PBS to remove the culture dish mechanically to use for the following *in vitro* experiments. This animal experiment was performed in accordance with the Guidelines for Proper Conduct of Animal Experiments of the Science Councils of Japan and approved by the Animal Research Control Committee of Okayama University (approval no. OKU-2016277).

#### Mouse Cranial Crown–Derived Cells (MC3T3-E1 Cells)

MC3T3-E1 cells (ATCC, Manassas, VA, United States) were used as pre-osteoblasts. MC3T3-E1 cells were cultured for 3 days in culture dishes containing DMEM (Wako) supplemented with 10% FBS (Invitrogen) and antibiotics (0.2 mg/ml gentamicin) (Gibco). Osteogenic differentiation was investigated by supplementing media with 50 mg/L ascorbic acid, 10 μM hydrocortisone, and 10 mM β-glycerophosphate (Takara Bio, Shiga, Japan) (Aaron et al., 2010).

### Western Blotting

mBMMs (1.0 × 10^5^ cells/well) were harvested from a 12-well plate and treated with RANKL (100 ng/ml) and M-CSF (100 ng/ml) in the presence and absence of TER (10 μM) for 10–30 min (to detect PKCα, phospho-PKCα/βII, and phospho-PKCδ expression). After each time course, cells were lysed rapidly by adding ice-cold cell lysis buffer containing 50 mM NaCl, 10 mM Tris-HCl (pH 7.2), 1% Nonidet P-40, 5 mM EDTA-Na, 1 mM sodium ortho vanadate, 1% sodium dodecyl sulfate (SDS), and a protease inhibitor cocktail (Sigma-Aldrich) for 10 min, according to a previously described method (Omori et al., 2004). Protein concentration was determined using a Bradford assay by using bovine serum albumin (BSA, Sigma-Aldrich) as the standard (Bradford, 1976). The lysates (30 μg) were mixed with SDS sample buffer (1% [w/v] SDS, 45 mM Tris-HCl [pH 6.8], 15% [v/v] glycerol, 144 mM 2-mercaptoethanol, and 0.002% bromophenol blue), and samples were boiled for 5 min. The samples were separated using SDS-polyacrylamide gel electrophoresis (PAGE) on a 7.5% or 12% polyacrylamide slab gel and transferred onto polyvinylidene difluoride membranes. These membranes were blocked with 5% skim milk in Tris-buffered saline with Tween 20® (TBST; 20 mM Tris-HCl (pH 7.6) containing 150 mM NaCl and 0.1% (v/v) Tween 20®) for 1 h and incubated with anti–phospho-PKCα/βII (1:1,000) and anti-total PKCα (1:1,000) overnight at 4°C. The membranes were incubated with secondary antibodies (goat anti-rabbit immunoglobulin G (IgG)–horseradish peroxidase (HRP) conjugate, 1:2,000 dilution; GE Healthcare, Chicago, IL, United States) for 1 h. HRP activity was visualized by incubating the membranes and using an electrochemiluminescence (ECL) detection system (SuperSigna® West Dura Extended Duration Substrate, Thermo Fisher Scientific), followed by autoradiography. At the end of these experiments, the immunodetection system and the bound antibody were removed from the blots by incubating the membranes with re-probing buffer (Restore™ Western Blot Stripping Buffer, Thermo Fisher Scientific). The blots were then stained with anti–β-actin antibody (1:2,000 dilution) to confirm that equal amounts of protein were present in each lane of the gel.

### Real-Time Polymerase Chain Reaction

mBMMs (1.0 × 10^5^ cells/well) were harvested from a 12-well plate and treated with RANKL and M-CSF (100 ng/ml each) in the presence and absence of TER (10 μM) for 2 days. Total ribonucleic acid (RNA) was then isolated using the RNeasy® Mini Kit (Qiagen, Hilden, Germany), followed by deoxyribonucleic acid (DNA) removal using an RNase-free DNase Kit (Qiagen). A total of 1 μg of high-quality total RNA was then reverse-transcribed using the SuperScript® III First-Strand Synthesis System (Thermo Fisher Scientific, Waltham, MA, United States). Amplification reactions were performed using SYBR® Green PCR Master Mix (Thermo Fisher Scientific). Up to 1 ng of complementary DNA was then amplified using specific primers. The reactions were performed using a 7,300 Real-Time PCR System (Thermo Fisher Scientific). The ratios of messenger RNA levels to control values were calculated using the ΔCt method (2^−ΔΔCt^). All data were normalized to the housekeeping control gene, β-actin. The PCR conditions used were as follows: 10 min at 95°C, followed by 40 cycles of 15 s at 95°C, and 60 s at 60°C. The primers used were as follows: 5′-TGG​GCC​TCC​ATA​TGA​CCT​CGA​GTA​G-3′ (forward) and 5′-TCA​AAG​GCT​TGT​AAA​TTG​GAG​GAG​T-3′ (reverse) for osteoclast stimulatory transmembrane protein (*Ocstamp*), 5′-CTA​GCT​GGC​TGG​ACT​TCA​TCC-3′ (forward) and 5′-TCA​TGC​TGT​CTA​GGA​GAC​CTC-3′ (reverse) for dendritic cell–specific transmembrane protein (*Dcstamp*), 5′-TGC​AGA​CAA​CTC​TTG​GTT​GG-3′ (forward) and 5′-TCG​GTT​TCT​TCT​CCT​CTG​GA-3′ (reverse) for calcitonin receptor (*Calcr*), 5′-TCA​GAT​CTC​TTC​AAG​GCT​GTG​CTG-3′ (forward) and 5′-GTG​CCA​AAT​GAG​TTC​AGA​GTG​ATG-3′ (reverse) for v-type protein ATPase subunit d2 (*Atp6v0d2*), 5′-CTG​CTG​GTA​ACG​GAT​CAG​CTC​CCC​AGA-3′ (forward) and 5′-CCA​AGG​AGC​CAG​AAC​CTT​CGA​AAC​T-3′ (reverse) for osteoclast-associated receptor (*Oscar*), 5′-ATG​CCA​GCG​ACA​AGA​GGT​TC-3′ (forward) and 5′-TGG​TTT​CCA​GCC​AGC​ACA​TAC-3′ (reverse) for *Trap* (*Acp5*), 5′- TGA​CCA​CTG​CCT​TCC​AAT​ACG-3′ (forward) and 5′-TGC​ATT​TAG​CTG​CCT​TTG​CC-3′ (reverse) for *cathepsin-K*, 5′-TGT​GTG​CCT​GGT​GCT​CAG​A-3′ (forward) and 5′-AGC​AGG​TTC​TCC​TTC​AGG​TTA​CA-3′ (reverse) for integrin β3 (*Itgb3*), 5′-CCA​GTC​AAG​AGC​ATC​AGC​AA-3′ (forward) and 5′-AAG​TAG​TGC​AGC​CCG​GAG​TA-3′ (reverse) for Proto-oncogene c-fos (*c-fos*), and 5′-TAG​CGG​AAC​CGC​TCA​TTG​CC-3′ (forward) and 5′-TTC​ACC​CAC​ACT​GTG​CCC-3′ (reverse) for *β-actin* (Kwak et al., 2010; Choi et al., 2012; Lee et al., 2012; Ma et al., 2014; Shimada-Sugawara et al., 2015; Nakagawa et al., 2020).

### Alizarin Red Staining and Alkaline Phosphatase Staining

MC3T3-E1 cells (1.0 × 10^5^ cells/well) were cultured onto 48-well plates, and treated with DMEM (Wako) supplemented with 10% FBS (Invitrogen), antibiotics (0.2 mg/ml gentamicin) (Gibco), 50 mg/L ascorbic acid, hydrocortisone, and 10 mM β-glycerophosphate (Takara Bio, Shiga, Japan) in the presence and absence of TER (0.1–10 μM) and recombinant mouse bone morphogenetic protein (BMP)-2 (50 ng/ml) (BioLegend, San Diego, CA, United States) for 21 days. Prior to staining, all cells were washed with PBS and fixed with 4% paraformaldehyde (PFA). Then, the cells were stained with an Alizarin red staining kit (Cosmo Bio Co., Ltd., Tokyo, Japan) and an ALP Staining Kit (Fuji Films Co., Tokyo, Japan), following the manufacturer’s instructions (Aaron et al., 2010).

### Statistical Analysis

Experimental results are presented as the mean ± standard deviation (SD). Multiple comparisons were conducted using one-way analysis of variance (ANOVA) and Tukey’s test. Statistical analysis software GraphPad Prism8 (GraphPad Software Inc., San Diego, CA, United States) was used for each statistical process, and *p*-values lower than 0.05 were considered statistically significant.

## Results

### Synthetic-(+)-Terrein–Suppressed Ovariectomy-Induced Systemic Bone Loss

**FIGURE 3:**
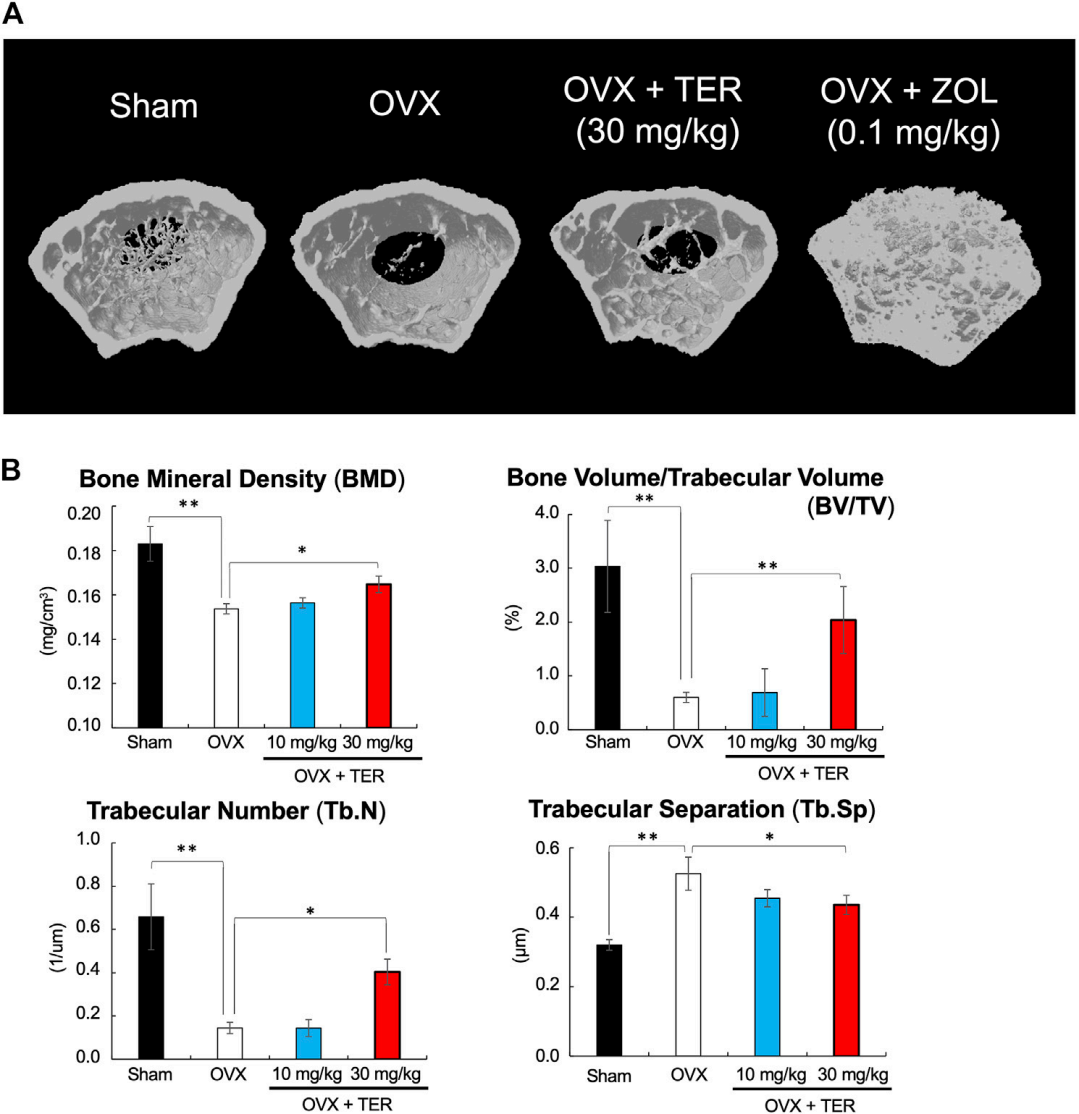
Synthetic TER suppressed ovariectomy-induced bone loss. **(A)** The 3D model of the femur was created within 3 mm just below the growth plate (the preferred site of femoral fracture). **(B)** Bone mineral density (BMD, mg/cm^3^), bone volume/trabecular volume (BV/TV, %), trabecular number (Tb.N, 1/μm), and trabecular separation (Tb.Sp, μm) were analyzed in each group (*n* = 6–7, **p < 0.01, *p < 0.05, ANOVA/Tukey–Kramer test). Sham: PBS-treated non-osteoporosis mice; OVX: PBS-treated osteoporosis mice; OVX + TER: TER-treated osteoporosis mice.

**FIGURE 4:**
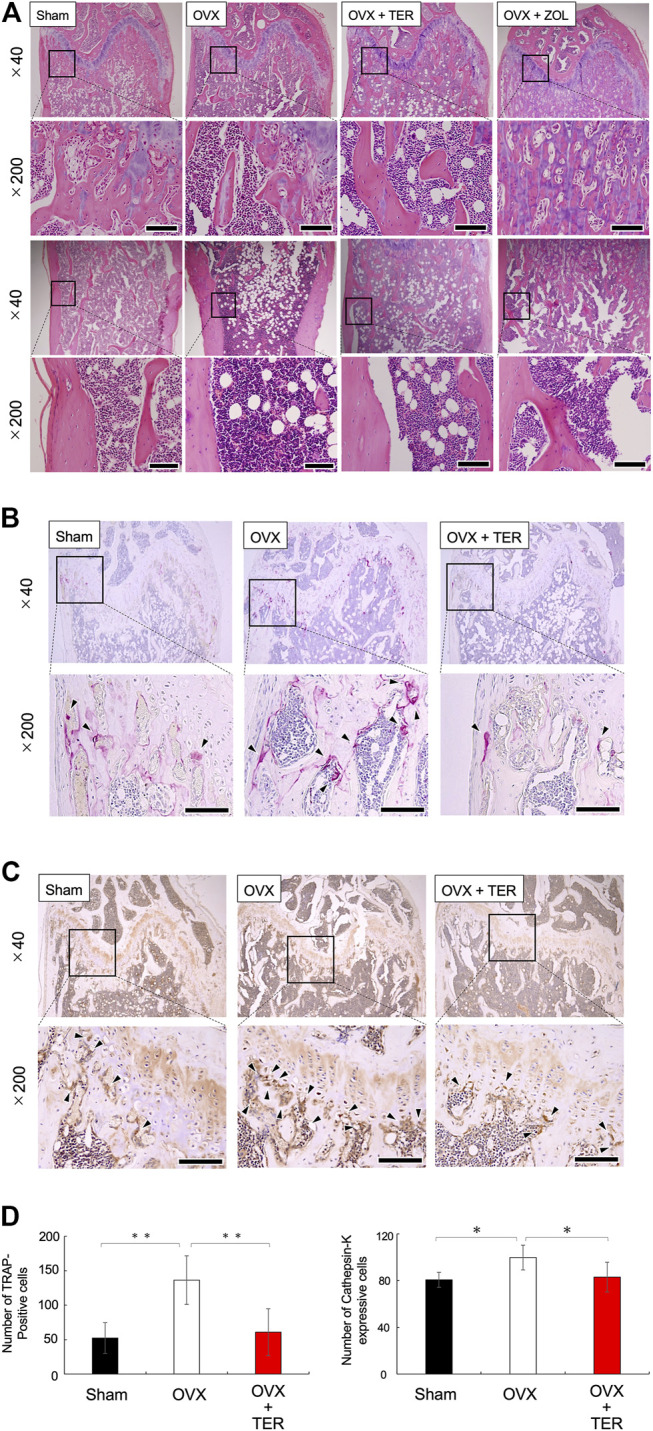
Synthetic TER suppressed osteoclast differentiation. Representative histological images of **(A)** HE-stained, **(B)** TRAP-stained (osteoclast), and **(C)** anti–cathepsin-K antibody–stained cells in each group are shown. The enlarged image of the area enclosed in the black line frame of the HE-stained image is shown in the lower row. Numbers next to the images indicate the magnification; scale bar: 500 µm (at a ×40 magnification) and 200 µm (at a ×200 magnification). **(D)** The number of osteoclasts on the femur bone was measured (*n* = 3, mean ± SD, **p < 0.01, ANOVA/Tukey–Kramer test) by defining the osteoclasts as cells that were TRAP positive and which had more than three nuclei. The number of positive cathepsin-K–expressing cells on the femur was measured (*n* = 3–4, mean ± SD, *p < 0.05, ANOVA/Tukey–Kramer test) by defining positive cells as those with darker cathepsin-K staining around the cell nucleus. Error bars: SD; dots: measured values; PBS: phosphate-buffered saline; Sham: PBS-treated non-osteoporosis mouse; OVX: PBS-treated osteoporosis mouse; OVX + TER: TER-treated osteoporosis mouse.

OVX models were used to validate the therapeutic value of TER in suppressing osteoporosis *in vivo*. TER or PBS (vehicle) was intraperitoneally injected into OVX mice every 2 days for 8 weeks before euthanization. Micro CT analyses were performed to determine the effect of synthetic TER on bone mass (Figure 3A). Compared to that of the Sham group, BMD, BV/TV, and Tb.N in the OVX + vehicle group decreased by approximately 16, 80, and 78%, respectively, whereas Tb/Sp increased by 64%. Interestingly, compared to the OVX group, after treatment with synthetic TER (30 mg/kg), BMD, BV/TV, and Tb.N increased by 7, 239, and 130%, respectively, whereas the relative Tb.Sp decreased by 42% (Figure 3B, *n* = 6–7, **p < 0.01, *p < 0.05). On the other hand, an abnormal increase in bone mass was observed in the ZOL-treated group (Figure 3A). Histomorphometric assessments (HE staining) validated these results (Figure 4A). Furthermore, TRAP and IHC staining were performed for qualitative and quantitative analyses of several bone parameters that reflect the effect of synthetic TER on osteoclast formation *in vivo* (Figures 4B,C). The number of TRAP-positive osteoclasts and cathepsin-K–positive cells decreased, together with a dramatic inhibition of osteoclast formation under treatment with synthetic TER, compared to the OVX + vehicle group (Figures 4B–D, *n* = 3, **p < 0.01, *p < 0.05).

### Synthetic-(+)-Terrein did Not Affect Body Weight and Serum Inflammatory Cytokines Production in Ovariectomized Mice

**FIGURE 5:**
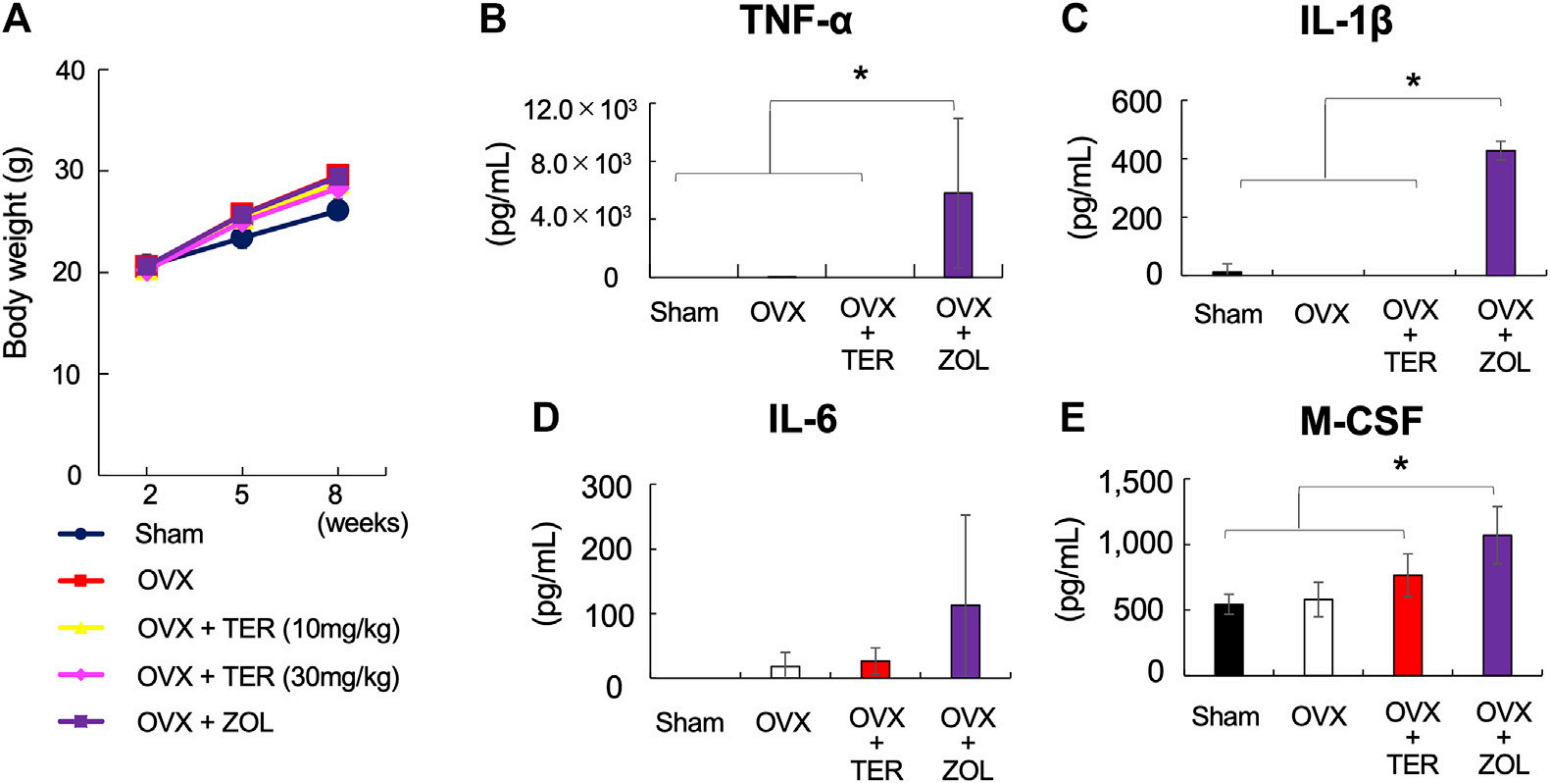
Synthetic TER did not affect the body weight and serum inflammatory cytokines production. **(A)** Effect of synthetic TER on body weight. Weight changes in Sham, OVX, OVX + TER (10–30 mg/kg, two times a week), and OVX + zoledronic acid (ZOL, 0.1 mg/kg, two times a week) groups were observed up to 2, 5, and 8 weeks after the start of the experiment as relative values (mean ± SD) of the baseline body weight value (100%) at the start of the experiment (*n* = 4–7). The error bar: SD. **(B–E)** The amount of pro-inflammatory cytokines (TNF-α, IL-1β, IL-6, and M-CSF) in the serum was quantified using ELISA (*n* = 4–7). Columns represent the mean results of the experiments carried out in, at least, triplicates, and the bars represent the SD (*n* = 4–7, *p < 0.05, ANOVA/Tukey–Kramer test).

There were no significant differences in body weight between the groups treated with/without synthetic TER (30 mg/kg) (Figure 5A). The serum inflammatory cytokine levels of TNF-α, IL-1β, IL-6, and M-CSF in the OVX mice did not differ between the groups treated with/without synthetic TER (30 mg/kg). However, in the ZOL-treated group, the secretion of inflammatory cytokines (TNF-α, IL-1β, IL-6, and M-CSF) was significantly enhanced (Figures 5B–E, *n* = 4–7, *p < 0.05).

### Synthetic-(+)-Terrein Inhibited the Phosphorylation of PKCα/βII and Receptor Activator of Nuclear Factor-kB Ligand–Induced Osteoclastogenesis in Mouse Bone Marrow Macrophages

**FIGURE 6:**
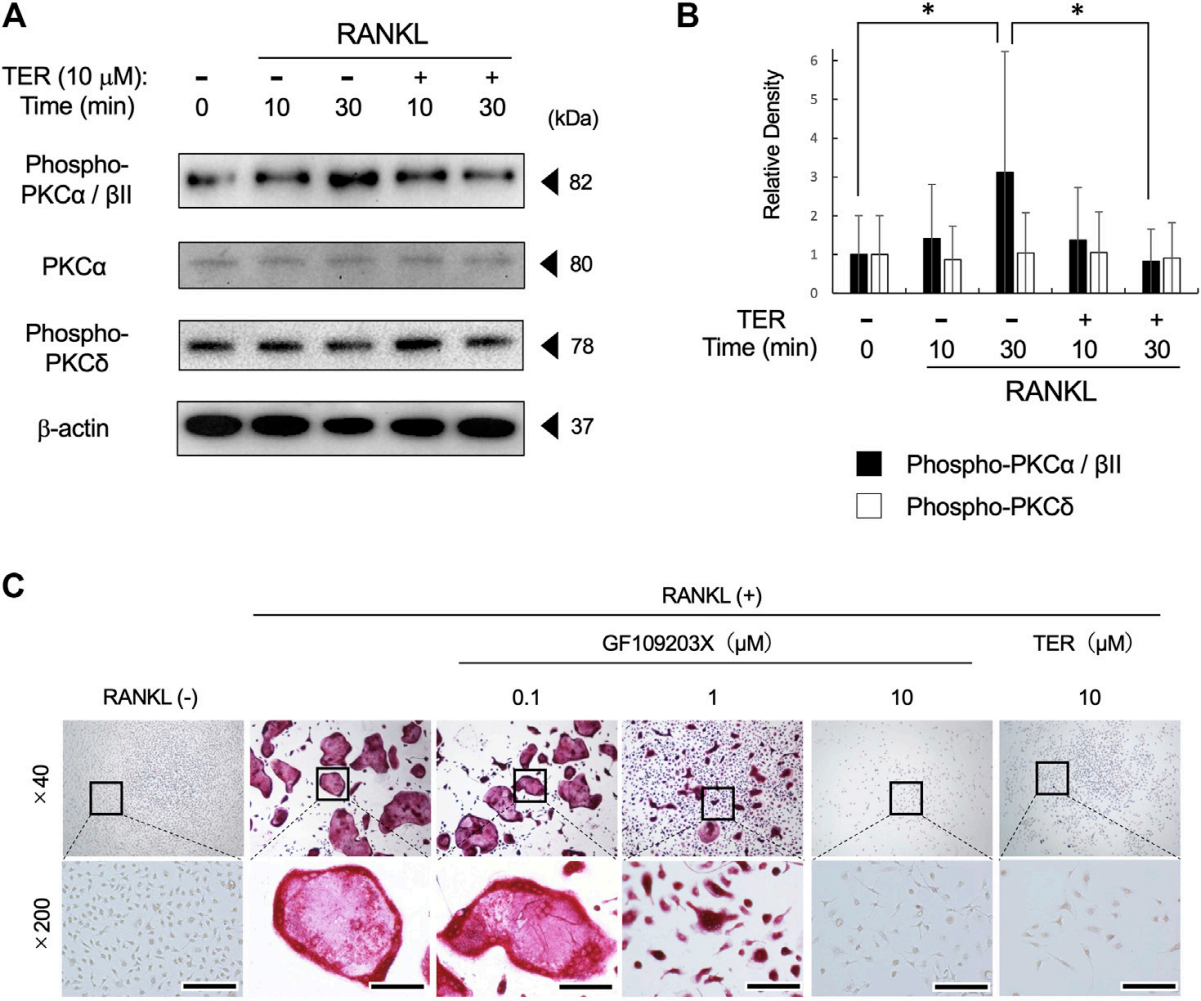
Synthetic TER suppressed RANKL-induced PKCα/βII phosphorylation and RANKL-induced osteoclastogenesis. **(A)** Mouse BMMs (1 × 10^5^ cells/well) were treated with RANKL/M-CSF (100 ng/ml each) in the presence and absence of synthetic TER (10 μM) for 30 min before RANKL stimulation. Total cell extracts were prepared and subjected to Western blotting using anti–phospho-PKCα/βǁ, total-PKCα, phosphor-PKCδ, and β-actin. **(B)** The relative density was measured using ImageJ software. Columns represent the mean results of the experiments carried out in triplicates, and the bars represent the SD (*n* = 3, *p < 0.05, ANOVA/Tukey–Kramer test). **(C)** Representative images of a 48-well plate showing the effects of the PKC inhibitor, GF102903X (0.1–10 μM), and synthetic TER (10 μM) on RANKL-induced mBMM-derived osteoclast-like cell formation stained with tartrate-resistant acid phosphate (TRAP). Numbers next to the images indicate the magnification; scale bar: 200 µm (at a ×200 magnification).

To elucidate the potential mechanism by which TER inhibits RANKL-induced osteoclastogenesis, we focused on the PKC signaling pathway, which is also related to NFATc1 expression on RANK–RANKL signaling. RANKL stimulation strongly induced PKCα/βII protein phosphorylation at 30 min. The treatment with synthetic TER (10 μM) significantly inhibited RANKL-induced PKCα/βII protein phosphorylation, which is related to NFATc1 expression (Figures 6A,B, *n* = 3, *p < 0.05). On the other hand, TER did not suppress PKCδ protein phosphorylation, which is not related to NFATc1 expression (Figures 6A,B, *n* = 3). In addition, an osteoclastogenesis assay was performed to investigate the effect of the PKC inhibitor, GF109203X, and synthetic TER. Treatment with GF109203X resulted in the dose-dependent inhibition of RANKL-induced osteoclast formation, especially in the 10 μM GF109203X-treated group, as well as GF109203X; synthetic TER (10 μM) inhibited RANKL-induced osteoclastogenesis in mBMMs completely (Figure 6C).

### Synthetic-(+)-Terrein Suppressed Receptor Activator of Nuclear Factor-kB Ligand–Induced mRNA Expression of Osteoclast Marker Genes in Mouse Bone Marrow Macrophages

**FIGURE 7:**
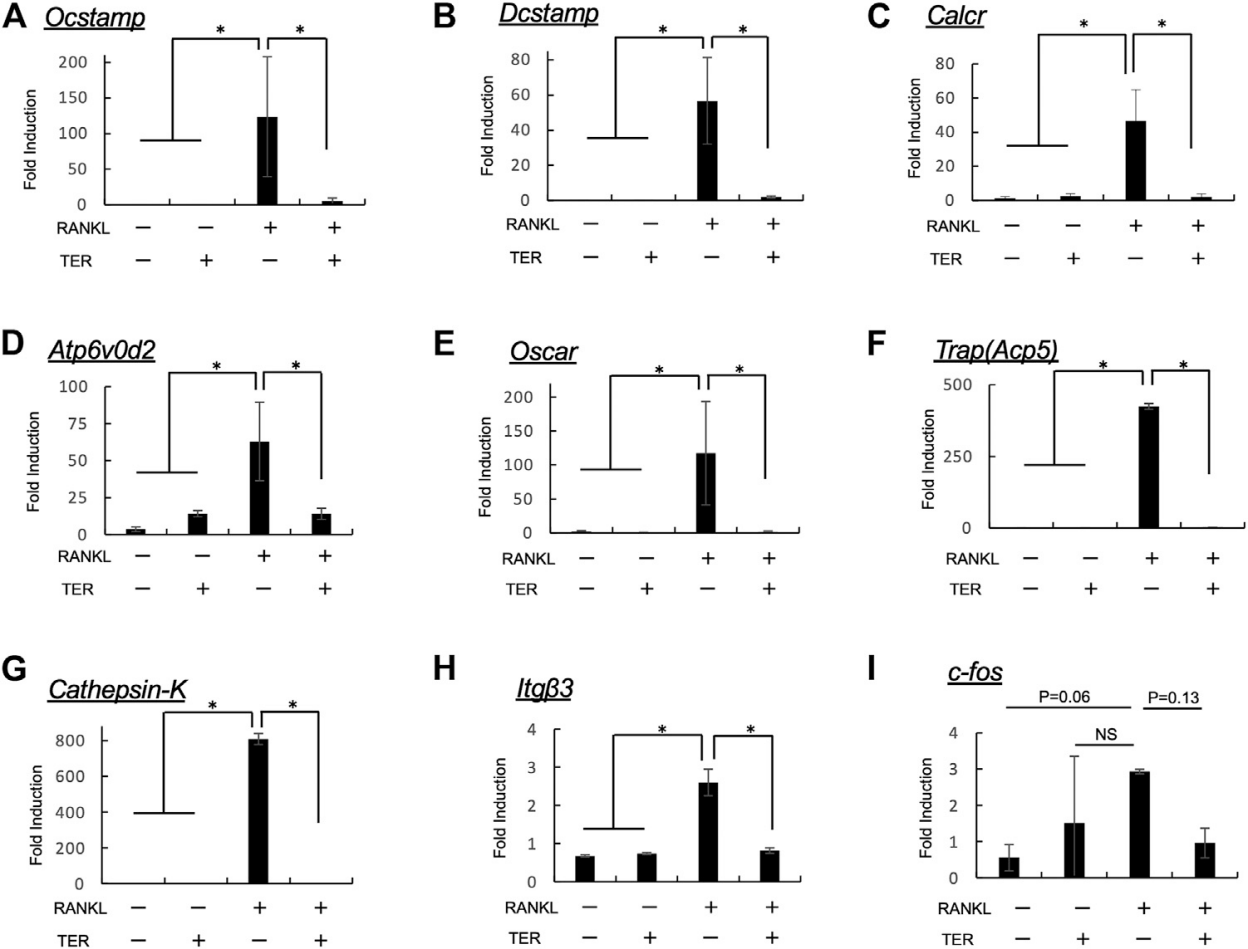
Synthetic TER inhibited RANKL-induced mRNA expression of osteoclastogenesis-related factors. Mouse BMMs (1 × 10^5^ cells/well) were incubated in serum-free medium containing RANKL/M-CSF (100 ng/ml each) in the presence and absence of synthetic TER (10 μM) for 48 h. Cells were lysed, and the total RNA was subjected to real-time reverse transcription–polymerase chain reaction (RT-PCR) to determine *Ocstamp*, *Dcstamp*, *Calcr*, *Atp6v0d2*, *Oscar*, *Trap(Acp5)*, *Cathepsin-K*, *Itgb3*, and *c-fos* gene expression levels. The columns represent the mean relative intensity of **(A)** *Ocstamp*, **(B)** *Dcstamp*, **(C)** *Calcr*, **(D)** *Atp6v0d2*, **(E)** *Oscar*, **(F)** *Trap(Acp5)*, **(G)** *Cathepsin-K*, **(H)** *Itgb3*, and **(I)** *c-fos* compared to that of *β-actin* carried out in triplicates, and bars represent the SD (*n* = 3, *p < 0.01, ns: not significant, ANOVA/Tukey–Kramer test).

To confirm the effect of TER on the expression of osteoclast function–related genes, *Ocstamp*, *Dcstamp*, *Calcr*, *Atp6v0d2*, *Oscar*, *Trap (Acp5)*, *Cathepsin-K, Itgb3*, and *c-fos* were determined. Compared to the control group, the expression of *Ocstamp*, *Dcstamp*, *Calcr*, *Atp6v0d2*, *Oscar*, *Trap (Acp5)*, *Cathepsin-K*, and *Itgb3*, but not of *c-fos*, was significantly suppressed by synthetic TER (10 μM, Figure 7, *n* = 3, **p < 0.01).

### Synthetic-(+)-Terrein did Not Affect Osteoblast Differentiation in MC3T3-E1 Cells

**FIGURE 8:**
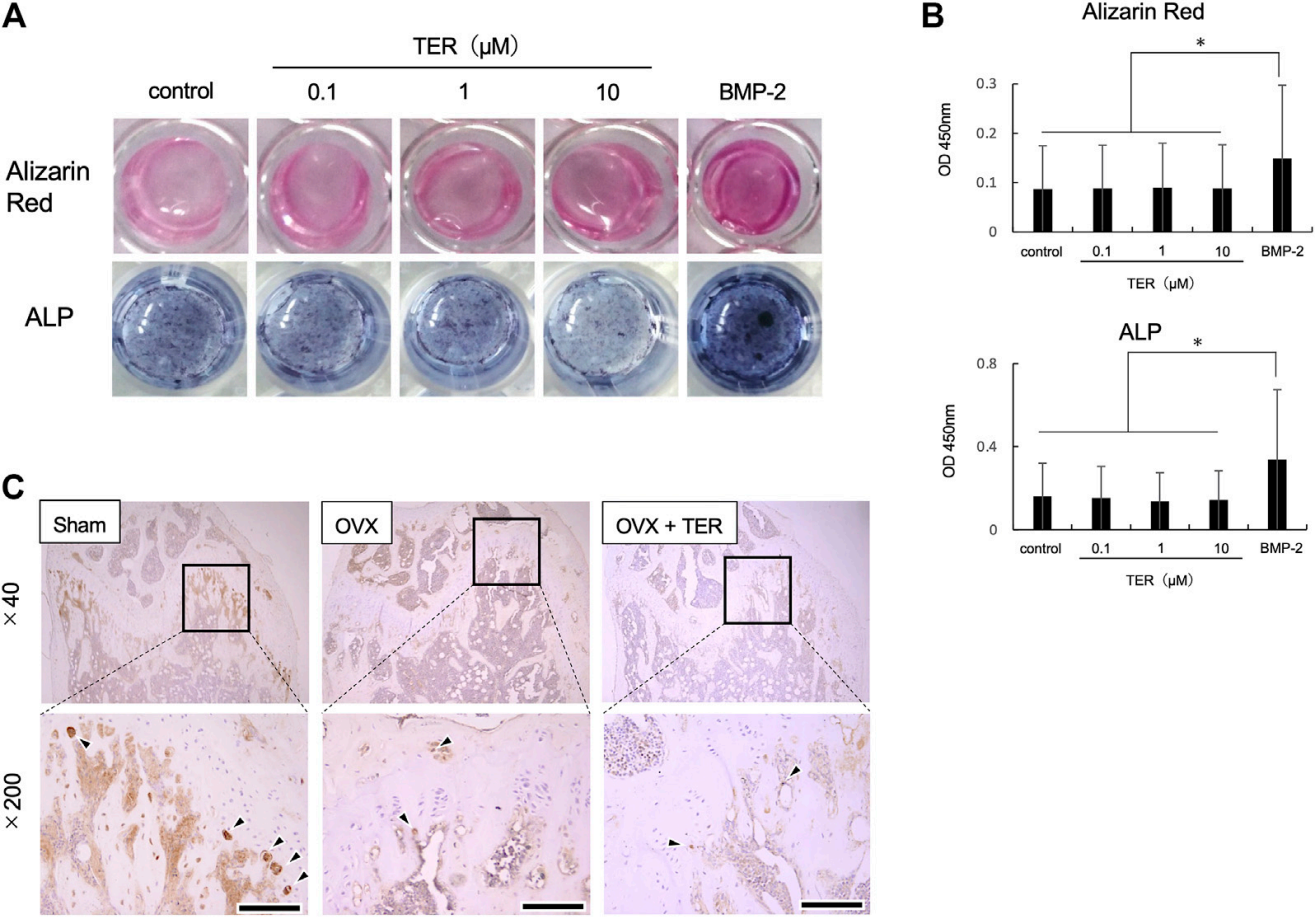
Synthetic TER did not affect osteoblast differentiation. Mouse MC3T3-E1 cells (1.0 × 10^5^ cells/well) were treated with ascorbic acid (50 μg/ml) and hydrocortisone (10 μM)/β-glycerophosphate (10 mM)/bone morphogenetic protein 2 (BMP-2, 100 ng/ml) in the presence or absence of synthetic TER (0.1–10 μM) for 3 weeks. **(A)** Osteoblast-stained image, alizarin red staining, and ALP staining. **(B)** Evaluation of calcification and ALP activity (*n* = 3, mean ± SD, *p < 0.05, ANOVA/Tukey–Kramer test). **(C)** IHC-stained (anti-ALP antibody) cells.

Whether TER promotes osteoblast differentiation was also examined using mouse osteoblast-like cells, MC3T3-E1. As shown in Figure 8, TER treatment did not promote alizarin red staining and ALP staining activity, while BMP-2 enhanced both staining and activity (Figures 8A,B, *n* = 3; **p < 0.01). We also performed IHC staining using an ALP antibody in tissue sections extracted from OVX mice. There was no statistical difference in the number of ALP-positive cells in each group (Figure 8C, *n* = 3).

## Discussion

In this study, we demonstrated that synthetic TER is capable of suppressing RANKL-induced osteoclast differentiation by inhibiting signaling pathways (PKCα/βII) and downstream function-related gene expression (OC-stamp, DC-stamp, calcitonin receptor, and v-type protein ATPase subunit d2). An OVX mouse model further proved the therapeutic value of TER in systematic bone loss. The *in vitro* and *in vivo* results indicated that TER probably has therapeutic potential for osteolytic diseases, including osteoporosis.

Bone homeostasis is maintained by the continuous progression of bone formation by osteoblasts and bone resorption by osteoclasts (Matsuo and Irie, 2008; Matsuoka et al., 2014). Overactivated osteoclasts can induce many pathological and osteochondrotic diseases, such as postmenopausal osteoporosis, rheumatoid arthritis, lytic bone metastases, Paget's disease, and periodontitis (Zaidi, 2007; Wackhoure et al., 2009; Kumar et al., 2018). In osteoporosis, estrogen deficiency leads to elevated levels of RANKL and inflammatory factors, including IL-6 and TNF-α (Lin et al., 2019). The overactivation of the RANKL signaling pathway recruits RANK/TRAF6 association, which in turn promotes osteoclast reproduction (Chen et al., 2018). Thus, inhibition of osteoclast formation in the RANKL signaling pathway makes it a therapeutic target for bone-destroying diseases.

In the present *in vivo* study, administration of synthetic TER maintained the bone mineral density, bone mass, and bone girdle count in OVX mice. Administration of ZOL, one of the BPs, enhanced the secretion of inflammatory cytokines in the serum, including TNF- α, IL-1β, IL-6, and M-CSF (Figure 5). On the other hand, administration of synthetic TER did not enhance the secretion of inflammatory cytokines in the serum; it had a biosafety impact compared to ZOL. Therefore, synthetic TER may be a safer treatment for osteoporosis than the currently used BPs.

NFATc1 plays an indispensable role in RANKL-induced osteoclast differentiation (Asagiri and Takayanagi, 2007). NFATc1 functions downstream of NF-κB, MAPKs (ERK1/2, p38, and MAPK), and Ca^2+^/calcineurin signaling pathways (Miyazaki et al., 2000; Takayanagi et al., 2002; Liu et al., 2007; Negishi-Koga and Takayanagi, 2009), and its activation can stimulate the expression of bone resorption–related genes, including *Atp6v0d2* (V-ATPase-d2), *Itgb3* (integrin 3), *Acp5* (TRAP), and *Ctsk* (cathepsin-K), which are essential for osteoclast differentiation (Crotti et al., 2008). Consistent with our findings, TER inhibits RANKL-induced osteoclast differentiation and bone resorption by inhibiting the expression of NFATc1 and its downstream bone resorption–related genes. On the other hand, TER did not suppress RANKL-induced phosphorylation of NF-κB, ERK, and p38 in mBMMs (Nakagawa et al., 2020). In this study, synthetic TER inhibited the RANKL-induced phosphorylation of PKCα/βII, one of the classical PKC pathways, and the PKC inhibitor, GF109203X, completely inhibited RANKL-induced osteoclastogenesis as well as synthetic TER treatment (Figure 6C). Previous studies have found that the PKCβ pathway, leading to GSK-3β inactivation and NFATc1 induction, has a key role in the RANKL-induced osteoclast differentiation (Shin et al., 2014). In addition, the PKC inhibitor, GF109203X, suppresses RANKL-induced calcium oscillations, inhibiting calcium-dependent NFAT activation in mBMMs (Yao et al., 2015). It is well known that NFATc1 levels are regulated by transcriptional control and by the actions of the Ca^2+^-dependent phosphatase calcineurin, which stabilizes the protein and allows its nuclear transportation and transcriptional activity (Negishi-Koga and Takayanagi, 2009). Thus, the mechanism by which TER inhibits osteoclast differentiation by suppressing PKCα/βII phosphorylation, but not NF-κB or MAPKs, was revealed, which may lead to the elucidation of a new mechanism of TER for inhibiting osteoclast differentiation *via* suppressing Ca^2+^ oscillation and GSK-3β dynamics that require further investigation.

For the future pharmacological use of TERs, the efficacy of synthetic TERs needs to be proven in comparison to existing osteoporosis drugs, such as BPs. The long half-life of BPs is one of the problems in osteoporosis treatment for MRONJ. An excessive increase in bone hardening also conversely increases the risk of fracture. Synthetic TERs are small molecules and, therefore, may have a shorter half-life than BPs. They are also expected to provide a moderate inhibitory effect on bone resorption as a treatment for patients with early osteoporosis. Therefore, further studies in an *in vivo* model are needed to elucidate the systemic response of TERs.

**FIGURE 9:**
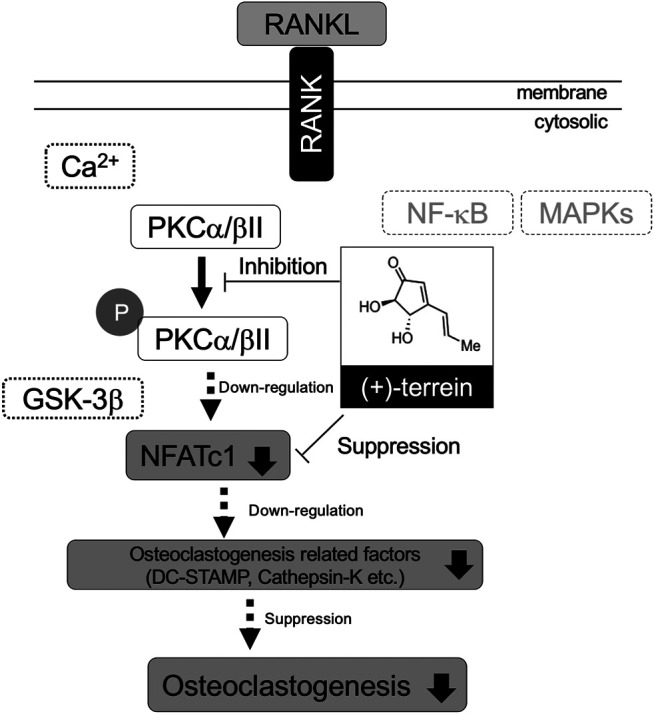
Schematic representation of the effect of TER on RANKL-induced osteoclastogenesis *via* suppressing PKCα/βII phosphorylation.

The present study shows that synthetic TER abrogates ovariectomy-induced bone loss and RANKL-induced osteoclastogenesis by suppressing PKCα/βII phosphorylation (Figure 9). The results provide new insights into the potential use of TER as an antiresorptive agent in the treatment of osteolytic bone diseases, including osteoporosis.

## Data Availability

The original contributions presented in the study are included in the article/Supplementary Material; further inquiries can be directed to the corresponding author.

## Glossary

TER: (+)-terrein
RANKL: receptor activator of nuclear factor-kB ligand
OVX: ovariectomized
mBMMs: mouse bone marrow macrophages
BPs: bisphosphonates
MRONJ: medicine-induced osteonecrosis of the jaw
TNF-α: tumor necrosis factor
IL: interleukin
VEGF: vascular endothelial growth factor
M-CSF: macrophage colony–stimulating factor
JAK-1: Janus-activated kinase
NFATc1: nuclear factor of activated T cell c1
NMR: nuclear magnetic resonance
IR: infrared
ZOL: zoledronic acid
PBS: phosphate-buffered saline
μCT: micro-computed tomography
BMD: bone mineral density
BV/TV: bone volume/trabecular volume
Tb.N: trabecular number
Tb.Sp: trabecular separation
TRAP: tartrate-resistant acid phosphatase
IHC: Immunohistochemical
DAB: 3,3′-diaminobenzidine
ELISA: enzyme-linked immunosorbent assay
FBS: fetal bovine serum
SDS: sodium dodecyl sulfate
BSA: bovine serum albumin
PAGE: polyacrylamide gel electrophoresis
TBST: Tris-buffered saline with Tween 20®
IgG: immunoglobulin G
HRP: horseradish peroxidase
ECL: electrochemiluminescence
PCR: polymerase chain reaction
ALP: Alkaline phosphatase
BMP: bone morphogenetic protein
PFA: paraformaldehyde
SD: standard deviation
ANOVA: analysis of variance
